# Sticky Floor, Broken Ladder, and Glass Ceiling in Academic Obstetrics and Gynecology in the United States and Canada

**DOI:** 10.7759/cureus.22535

**Published:** 2022-02-23

**Authors:** Katherine Y Kim, Emily L Kearsley, Hsin Yun Yang, John P Walsh, Mehr Jain, Laura Hopkins, Ahmad B Wazzan, Faisal Khosa

**Affiliations:** 1 Family Medicine, McMaster University, Hamilton, CAN; 2 Family Medicine, Queen's University, Kingston, CAN; 3 Radiology, Vancouver General Hospital, Vancouver, CAN; 4 Obstetrics and Gynecology, University of Ottawa, Ottawa, CAN; 5 Gynecologic Oncology, University of Saskatchewan, Saskatoon, CAN; 6 Obstetrics and Gynecology, King Abdulaziz University College of Medicin, Jeddah, SAU

**Keywords:** academic performance, obstetrics and gynecology department, gender bias, education department, leadership

## Abstract

Objective

To evaluate the gender proportion in academic obstetrics and gynecology faculty across the United States and Canada and further assess any gender differences in academic ranks, leadership positions, and research productivity.

Methods

Obstetrics and gynecology programs were searched from the Fellowship and Residency Electronic Interactive Database (FREIDA) (n=145) and the Canadian Resident Matching Service (CaRMS) (n=13) to compile a database of gender and academic profiles of faculty physicians with Medical Doctorate (MD) or Doctors of Osteopathic Medicine (DO) degrees. Elsevier's Scopus was used to gather individual research metrics for analysis, and the data were analyzed using Strata v14.2 (StataCorp. 2015. Stata Statistical Software: Release *14*. College Station, TX: StataCorp LP).

Results

Among 3556 American and 689 Canadian Obstetrics and Gynaecology physicians, women comprised 60.9% and 61.4%, respectively. Among physicians with professorships, women physicians comprised 36.2% and 35.8% in the United States and Canada, respectively. When examining the gender proportion of physicians in leadership roles, women comprised 52.2% and 56.1% in the United States and Canada, respectively. The h-index between men and women physicians showed a significant difference overall in both the United States (p<0.001) and Canada (p<0.001), indicating that men have higher academic output.

Conclusion

Although the overall proportion of women academic staff physicians in Obstetrics and Gynaecology is higher than the proportion of men, there are more men who had a full professor rank. Men also had higher academic productivity.

## Introduction

Over the last two decades, there have been deliberate efforts to bring about equity, diversity, and inclusion in academic medicine. As a result, the proportion of women in medicine has been steadily increasing. In the United States (US), women medical school graduates made up almost half of the total at 47.3% in 2017 [1]. In Canada in the same year, women physicians comprised 41.0% of total working physicians and the number of women physicians has increased by 19.2% from five years prior [2]. However, studies have shown that women physicians are still underrepresented in academic or leadership positions in various specialties and their professional organizations such as radiology [3-7], cardiology [8], and neurosurgery [9]. These studies also found an association with women physicians having less research productivity and lower h-indices, which suggests that gender bias in academic research is a factor intrinsically associated with gender bias in medical academia. As of 2017, Obstetrics and Gynaecology (OB/GYN) is one of the medical specialties that had a relatively higher representation of women physicians (57.9%, Canada; 58.7%, US) [10-11]. This proportion is on the rise, with women physicians comprising the majority of younger physicians in this specialty, with 62 women medical graduates in Canada matching to OB/GYN in 2017 as opposed to nine men medical graduates [12].

To promote gender equity among academic positions in medicine, it is important to understand the multiple factors that influence academic progression. For instance, greater domestic responsibility [13], lack of gender-specific role models in higher ranks [14], and the tendency of women physicians to choose clinical over academic career paths [15] could potentially be factors contributing to gender differences in higher academic ranks. Another factor that contributes to academic progression is research productivity, which is important for promotion to professorship positions [16]. The h-index, calculated as h, the greatest number of publications an author has that are cited at least h number of times, is a commonly used marker of research productivity. Although it cannot capture each individual’s research career accurately, the h-index shows a strong correlation with advancement in the academic career not only through promotion to higher faculty ranks but also through grant support for further research [16].

In this study, the objective was to determine the current gender proportion in academic OB/GYN faculty across both the US and Canada and further assess any gender differences in leadership positions and research productivity. A similar study looked at gender differences in the research productivity in the discipline of Gynecologic Oncology in the US and found that women physicians in this discipline had a lower h-index than men but only earlier in their career, namely, at the assistant professor level [17]. To our knowledge, there has been no study examining gender differences in the discipline of general OB/GYN. Furthermore, this study assesses and compares gender differences in the US and Canada. Our study complements and supplements the expanding research on gender differences in various medical specialties and provides a baseline to which researchers can compare future changes to gender proportions in medical academia.

## Materials and methods

This retrospective observational study used data collected from publicly available databases. The methodology used was validated through several publications and has been applied to study editorial boards, academic disciplines, professional societies, and NIH funding [3-6,8-9,18-23]. The list of medical schools with an OB/GYN residency program was obtained from Fellowship and Residency Electronic Interactive Database (FREIDA) and Canadian Resident Matching Service (CaRMS) for the US and Canada, respectively. There was a total of 274 OB/GYN programs from the US and 16 programs from Canada by the end of 2017. For each program, the list of its faculty physicians, their gender, and their academic position were obtained using the program’s official websites. Leadership roles were also collected if they were provided. Programs that did not provide all the information meeting the inclusion criteria were excluded. Brief personal profile statements on program websites and Google searches were used as complementary sources of missing profile information and to confirm the genders of the physicians. LinkedIn and Doximity served as complementary sources for obtaining missing information. Acceptable lists were obtained for 145 programs from US and 13 programs from Canada. No informed consent or ethics approval was required for this project, as only publicly available data were used.

The inclusion criteria for this study were the following: 1) current full-time academic faculty position in an OB/GYN department, and 2) Doctor of Medicine (MD) or Doctor of Osteopathic Medicine (DO) title. The eligible physicians were then searched on Elsevier's SCOPUS, a citation database, in order to obtain their information pertaining to academic productivity. These parameters include the number of publications, h-index, number of citations, and years of publication. We also looked at the proportion of men versus women physicians in leadership roles. Leadership roles were defined as any administrative position relevant to OB/GYN residency departments such as directors, coordinators, and chairs of the department.

The data were analyzed using Strata version 14.2 (StataCorp. 2015. Stata Statistical Software: Release 14. College Station, TX: StataCorp LP) and tested for normality using the Kolmogorov-Smirnoff test and histograms. The Mann-Whitney U test was used to test for significant differences between each academic parameters' h-index, number of publications, and number of citations of men and women faculty members at each academic position level.

## Results

**Figure 1:**
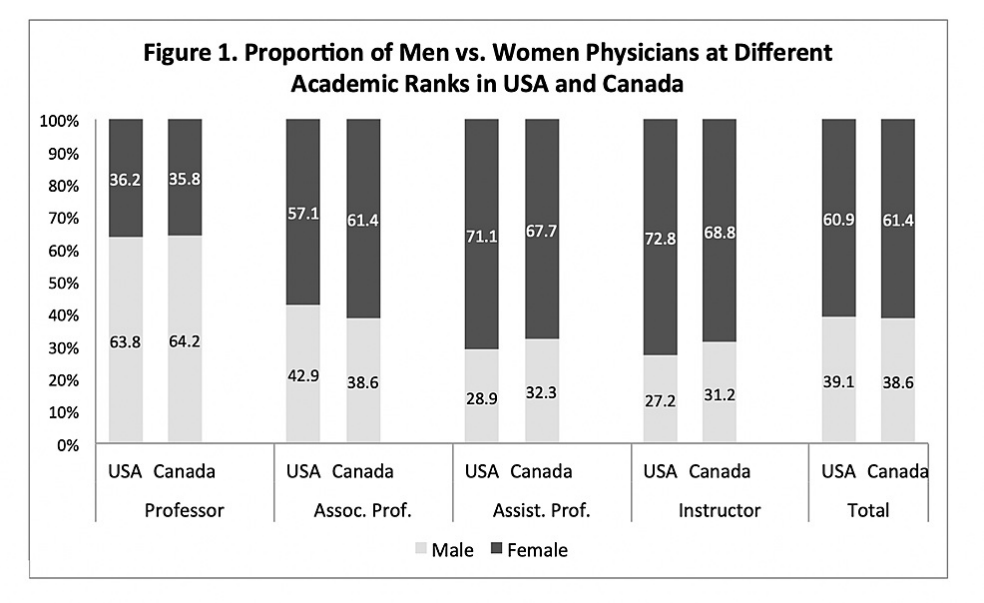
Proportion of men vs. women physicians at different academic ranks in medical education institutes of USA and Canada Academic ranks order from highest to lowest rank are professor, assoc. prof. (associate professor), assist. prof (assistant professor), and instructor. The total column shows the percentage of women and men physicians among physicians of all academic ranks combined.

**Figure 2:**
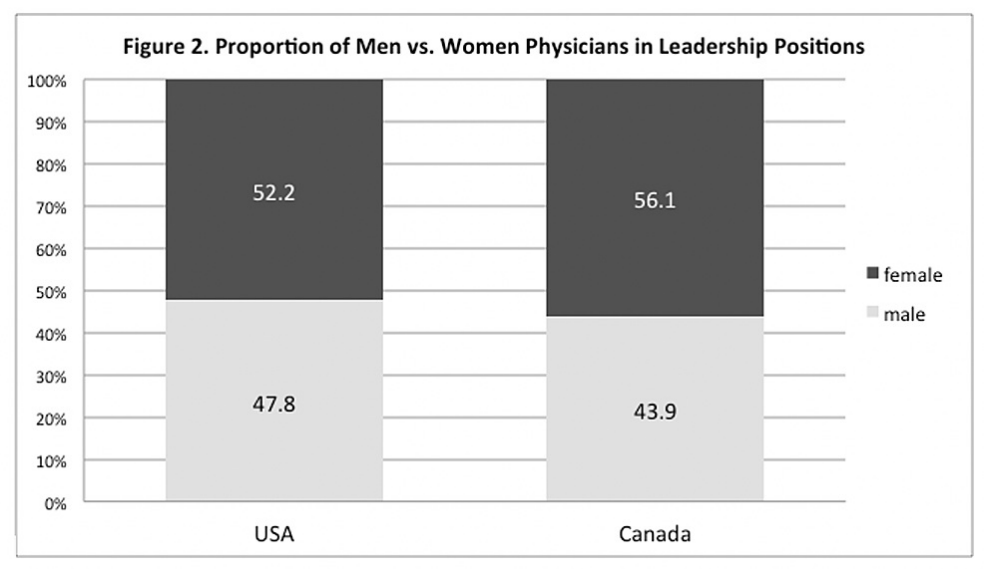
Proportion of men vs. women physicians in leadership positions in medical education institutes of USA and Canada Leadership positions include physicians with administrative roles in addition to their staff membership such as directors, coordinators, managers, etc.

Among the 3556 American and 689 Canadian physicians from academic OB/GYN departments, women physicians comprised 60.9% and 61.4% of the total, respectively (Table 1). However, the proportions decreased at higher academic ranks. Among the physicians with a full professor rank, women physicians comprised 36.2% and 35.8% in the US and Canada, respectively. Following this trend, physicians with lower academic ranks, such as at the instructor level, had a greater proportion of women (72.8%, US; 68.8%, Canada). The distribution of gender at each academic rank is shown in Figure 1. When examining the gender proportion of physicians in leadership roles, women comprised 52.2% and 56.1% of the US and Canada, respectively (Figure 2, Table 1).

**Table 1:** Proportion of men and women physicians at different academic ranks and leadership positions in numbers and percentages Abbreviations: N - number of physicians; % - percentage of physicians

Academic Rank	Men (N)	Men (%)	Women (N)	Women (%)	Total (N)
USA					
Professor	471	63.8	267	36.2	738
Associate Professor	332	42.9	441	57.1	773
Assistant Professor	527	28.9	1294	71.1	1821
Instructor	61	27.2	163	72.8	224
Total	1391	39.1	2165	60.9	3556
Canada					
Professor	68	64.2	38	35.8	106
Associate Professor	66	38.6	105	61.4	171
Assistant Professor	103	32.3	216	67.7	319
Instructor	29	31.2	64	68.8	93
Total	266	38.6	423	61.4	689
Leadership Positions	Men (N)	Men (%)	Women (N)	Women (%)	Total (N)
USA	531	47.8	579	52.2	1110
Canada	65	43.9	83	56.1	148
Total	596	47.4	662	52.6	1258

To assess for any differences in academic productivity of women and men physicians, their median number of publications, median h-index, and median number of citations were examined. For the number of papers published, the median number of articles overall and at each academic rank was compared for any significant differences using the Mann-Whitney test (Table 2). The results showed that men physicians have a significantly higher median number of articles overall, both in the US (24 vs.10; p<0.001) and in Canada (21 vs. 9; p=0.001). However, when assessing each academic rank individually, a statistically significant difference in the number of papers published was only seen at the level of full professorship and associate professorship in the US. Canada did not demonstrate any significant differences at any of the individual academic ranks.

**Table 2:** Median of the total number of academic articles published by men and women physicians at different academic ranks with statistical significance calculated by the Mann-Whitney test Abbreviations: N - number of physicians

Academic Rank	Men Median (N)	Women Median (N)		Significance (p-value)	
USA					
Professor	75	50		*<0.001	
Associate Professor	18.5	16		*0.039	
Assistant Professor	6	6		0.316	
Instructor	6	2		0.052	
Overall	24	10		*<0.001	
Canada					
Professor	78.5	56		0.066	
Associate Professor	18	20		0.700	
Assistant Professor	9.5	6		0.057	
Instructor	5.5	2		0.139	
Overall	21	9		*<0.001	

Similar to the number of articles published, the h-index between men and women physicians showed a significant difference overall in both the US (10 vs. 5; p<0.001) and Canada (10 vs. 5; p<0.001) (Table 3), indicating that men physicians have higher academic output. The same trend was seen for the median number of citations (339 vs. 120.5, p<0.001, US; 438 vs. 112, p<0.001, Canada) (Table 4). These two parameters showed significant differences between women and men at all academic ranks in the US (Tables 3-4). In comparison, no significant difference was seen at each academic rank in Canada except for the h-index for professors (Tables 3-4). 

**Table 3:** Median of the h-index of men and women physicians at different academic ranks with statistical significance calculated by the Mann-Whitney test

Academic Rank	Men (Median)	Women (Median)	Significance (p-value)
USA			
Professor	22	18	*<0.001
Associate Professor	8	7	*0.014
Assistant Professor	4	3	*0.031
Instructor	4	2	*0.001
Overall	10	5	*<0.001
Canada			
Professor	24	17	*0.039
Associate Professor	10	8	0.382
Assistant Professor	5	3	0.072
Instructor	2.5	2	0.800
Overall	10	5	*<0.001

**Table 4:** Median of the total number of citations men and women physicians of different academic ranks have with statistical significance calculated by the Mann-Whitney test

Academic Rank	Men (Median)	Women (Median)	Significance (p-value)
USA			
Professor	1793	1249	*0.001
Associate Professor	324	248.5	*0.012
Assistant Professor	75	54	*0.014
Instructor	78	18.5	*0.029
Overall	438	112	*<0.001
Canada			
Professor	1892	1193	0.065
Associate Professor	336	263	0.967
Assistant Professor	97	55	0.156
Instructor	58.5	31	0.817
Overall	339	120.5	*<0.001

## Discussion

When comparing the men to women composition in academic OB/GYN, there is a clear trend showing that although OB/GYN is a women-dominant discipline overall, men predominate in higher academic positions. However, the possibility that this is just a reflection of the gender proportion in OB/GYN of the past cannot be ruled out. That is to say, the current professors entered the workforce when men predominated the medical school matriculants and hence the physician workforce. This is also supported by the fact that the gender proportion at lower academic ranks is more similar to the current overall gender proportion of OB/GYN staff in total. According to the 2017 statistics report by the American College of Obstetricians and Gynecologists, women physicians make up less than 30% of OB/GYN fellowship-trained physicians between the age of 60 and 69 years versus greater than 70% of fellowship-trained physicians between the age of 30 and 39 years in the US [10]. Likewise in Canada, 35.5% of 55 to 64-year-old physicians in OB/GYN are women physicians but women physicians make up 60.4% of working physicians under 35 years of age [2]. Since academic promotions are usually achieved in a stepwise progressive fashion, higher-ranking positions may potentially be held by older physicians whose gender ratio reflects the ratio of the past.

In comparison, the gender ratios for leadership roles are closer to half in both the US and Canada (47.8% vs. 52.2%, US; 43.9% vs. 56.1%, Canada; Table 1). Considering that OB/GYN is a women-dominant discipline, this still indicates a relatively greater representation of men physicians in higher leadership roles.

Multiple studies have demonstrated that medical academia tends to have greater male representation with higher academic productivity [3-4,7]. Being one of the women-dominant specialties, OB/GYN has more women physicians holding teaching positions than men physicians. However, the pattern of men physicians having higher research productivity still persists in OB/GYN as well. From the study findings, the US shows significant differences between men and women physicians for the number of articles published at the professor and associate professor levels (75 vs. 50, p<0.001) (Table 1). For h-index and number of citations, there is a significant difference at all academic ranks (Tables 2-3). Interestingly, Canada shows similar trends but with no statistical significance, which may be the result of a smaller sample size. However, at lower academic ranks, these differences in academic productivity can be minimal and have questionable practical significance. For instance, the h-index difference between men and women associate professors in the US was eight vs. nine (Table 3).

Hurdles confronted by women to attain leadership positions are complex and multidimensional, as no one single reason can be identified to be the most oppressive one. More apparent reasoning would be the ‘Pipeline’ phenomenon, which explains how women have recently increased in number in medical schools and residency programs and therefore require a few more years before they can be considered experienced and adept enough to take up leadership positions on academic and organizational fronts [24-25]. Other possible explanations for the lower academic productivity of women are the potential barriers that may hinder women physicians from accessing the same research opportunities as men. For instance, women researchers not having equal access to research grants [15,26] or the higher proportion of women physicians choosing clinical career paths rather than research paths [15] are factors associated with slower academic career progression.

Our study has its share of limitations, including that there is a margin of error with the quality of the publicly available data since it may not be updated. Another limitation is that the study does not include data regarding the physicians’ age, which may be a variable influencing the difference in the academic success of women and men physicians in OB/GYN. A limitation of this study is concerned with the use of the h-index to measure research or scholarly productivity. Papers can frequently be self-cited, and this may or may not intentionally inflate one’s h-index [27]. Also, one value, i.e. the h-index, is fruitless in understanding how different types of researchers and authorship positions impact one's scholarly productivity. Another limitation involves the use of Scopus in this study to extract h-indices. Authors with similar names can be mistaken while extracting data from Scopus so a Google search was done to verify names from curriculum vitae found on departmental and organizational websites. All women present in the study sample were also verified in this way so that ones who changed their surnames after marriage did not have distorted research productivity on Scopus. Another inevitable limitation of using the h-index is that it changes rapidly. The same limitation can be applied to the unplanned and planned repositioning of the academic and organizational rank of an individual that can take place during the time data were collected, analyzed, and, eventually, the article was sent for publishing. Therefore, the current article has data accurate as of January 2018. Lastly, a limitation of this study is that it only presents a status report. This study will be useful for future comparisons to see the change in academic accomplishments across genders in the coming years.

It is important to recognize gender underrepresentation in academic medicine in order to assess the underlying factors and plan remedial action. To support female physicians, local and national level organizations can develop committees to directly address the gender gap [28-29]. Organization subcommittees ensure that resources and time are allocated to improving representation in medicine while also creating spaces to continue the discussion of closing the gender gap in the workplace at large. Mentorship is another well-recognized tool to address the gender disparity in medicine [28]. For example, The American Association for Women Radiologists (AAWR) has produced successful faculty mentorship opportunities that have resulted in increased research productivity of members involved in the program [28,30]. Leaders should assess for gender differences in their departments in order to invest in and provide mentorship to facilitate equitable distribution of resources and leadership opportunities.

## Conclusions

In summary, although the overall proportion of women staff physicians in OB/GYN is higher than the proportion of men, there are more men who hold a full professor rank. Furthermore, men have a significantly greater number of published documents and number of citations and a higher h-index as compared to women in both the US and Canada, supporting that men physicians have higher academic productivity. Female physicians make up approximately half of the leadership positions in both the US and Canada. As a future step, assessing the proportion of men and women physicians at different stages of their academic careers in medicine in comparison to their age may provide a better understanding of the gender differences that are present today.

## References

[1] (2019). Total U.S. medical school graduates by race/ethnicity and sex, 2012-2013 through 2016-2017. Association of American Medical Colleges. https://www.aamc.org/download/321536/data/factstableb4.pdf.

[2] Physicians in Canada (2019). Physicians in Canada, 2017. Canadian Institute for Health Information. https://secure.cihi.ca/free_products/Physicians_in_Canada_2017.pdf.

[3] Ahmadi M, Khurshid K, Sanelli PC (2018). Influences for gender disparity in academic neuroradiology. AJNR Am J Neuroradiol.

[4] Battaglia F, Shah S, Jalal S (2019). Gender disparity in academic emergency radiology. Emerg Radiol.

[5] Hamidizadeh R, Jalal S, Pindiprolu B (2018). Influences for gender disparity in the radiology societies in North America. AJR Am J Roentgenol.

[6] Khurshid K, Shah S, Ahmadi M, Jalal S, Carlos R, Nicolaou S, Khosa F (2018). Gender differences in the publication rate among breast imaging radiologists in the United States and Canada. AJR Am J Roentgenol.

[7] Qamar SR, Khurshid K, Jalal S, Bancroft L, Munk PL, Nicolaou S, Khosa F (2018). Academic musculoskeletal radiology: influences for gender disparity. Skeletal Radiol.

[8] Khan MS, Usman MS, Siddiqi TJ (2019). Women in leadership positions in academic cardiology: a study of program directors and division chiefs. J Womens Health (Larchmt).

[9] Shaikh AT, Farhan SA, Siddiqi R, Fatima K, Siddiqi J, Khosa F (2019). Disparity in leadership in neurosurgical societies: a global breakdown. World Neurosurg.

[10] Rayburn WF (2019). The Obstetrician-Gynecologist Workforce in the United States: Facts, Figures and Implications, 2017. https://www.ncbi.nlm.nih.gov/nlmcatalog/101542193.

[11] (2019). Number and percent distribution of physicians by specialty and sex. Canadian Medical Association. https://www.cma.ca/sites/default/files/2019-03/2017-06-spec-sex.pdf.

[12] (2019). First choice discipline preference and match results of CMGs by gender, 2017. Canadian Resident Matching Service. https://www.carms.ca/wp-content/uploads/2018/05/table_19_first_choice_discipline_preference_and_match_results_of_cmgs_by_gender_english_2017.pdf..

[13] Jolly S, Griffith KA, DeCastro R, Stewart A, Ubel P, Jagsi R (2014). Gender differences in time spent on parenting and domestic responsibilities by high-achieving young physician-researchers. Ann Intern Med.

[14] Bickel J, Wara D, Atkinson BF (2002). Increasing women's leadership in academic medicine: report of the AAMC Project Implementation Committee. Acad Med.

[15] Jena AB, Khullar D, Ho O, Olenski AR, Blumenthal DM (2015). Sex differences in academic rank in US medical schools in 2014. JAMA.

[16] Bastian S, Ippolito JA, Lopez SA, Eloy JA, Beebe KS (2017). The use of the h-index in academic orthopaedic surgery. J Bone Joint Surg Am.

[17] Hill EK, Blake RA, Emerson JB (2015). Gender differences in scholarly productivity within academic gynecologic oncology departments. Obstet Gynecol.

[18] Battaglia F, Jalal S, Khosa F (2018). Academic general surgery: influences for gender disparity in North America. J Am Coll Surg.

[19] Ilin J, Langlois E, Jalal S, Khosa F (2020). Gender disparity within academic Canadian urology. Can Urol Assoc J.

[20] Moghimi S, Khurshid K, Jalal S, Qamar SR, Nicolaou S, Fatima K, Khosa F (2019). Gender differences in leadership positions among academic nuclear medicine specialists in Canada and the United States. AJR Am J Roentgenol.

[21] Shah A, Jalal S, Khosa F (2018). Influences for gender disparity in dermatology in North America. Int J Dermatol.

[22] Sheikh MH, Chaudhary AM, Khan AS, Tahir MA, Yahya HA, Naveed S, Khosa F (2018). Influences for gender disparity in academic psychiatry in the United States. Cureus.

[23] Yang HY, Rhee G, Xuan L, Silver JK, Jalal S, Khosa F (2019). Analysis of h-index in assessing gender differences in academic rank and leadership in physical medicine and rehabilitation in the United States and Canada. Am J Phys Med Rehabil.

[24] Amrein K, Langmann A, Fahrleitner-Pammer A, Pieber TR, Zollner-Schwetz I (2011). Women underrepresented on editorial boards of 60 major medical journals. Gend Med.

[25] Bismark M, Morris J, Thomas L, Loh E, Phelps G, Dickinson H (2015). Reasons and remedies for under-representation of women in medical leadership roles: a qualitative study from Australia. BMJ Open.

[26] Kaatz A, Lee YG, Potvien A (2016). Analysis of national institutes of health R01 application critiques, impact, and criteria scores: does the sex of the principal investigator make a difference?. Acad Med.

[27] Purvis A (2006). The h index: playing the numbers game. Trends Ecol Evol.

[28] Lebel K, Hillier E, Spalluto LB, Yap W, Keglowitsch K, Darras KE, Yong-Hing CJ (2021). The status of diversity in Canadian radiology—where we stand and what can we do about it. Can Assoc Radiol J.

[29] Golding PM (2015). Overcoming the gender gap: increasing gender diversity, scientific scholarship and social legitimacy of our profession. Australas Psychiatry.

[30] Spalluto LB, Arleo EK, Macura KJ, Rumack CM (2017). 35 years of experience from the American Association for Women Radiologists: increasing the visibility of women in radiology. J Am Coll Radiol.

